# Experimental Parameterisation of Principal Physics in Buoyancy Variations of Marine Teleost Eggs

**DOI:** 10.1371/journal.pone.0104089

**Published:** 2014-08-14

**Authors:** Kyung-Mi Jung, Arild Folkvord, Olav Sigurd Kjesbu, Svein Sundby

**Affiliations:** 1 Institute of Marine Research (IMR) and Hjort Centre for Marine Ecosystem Dynamics, Bergen, Norway; 2 Department of Biology, University of Bergen, Bergen, Norway; 3 Centre for Ecological and Evolutionary Synthesis (CEES), Department of Biology, University of Oslo, Oslo, Norway; Technical University of Denmark, Denmark

## Abstract

It is generally accepted that the high buoyancy of pelagic marine eggs is due to substantial influx of water across the cell membrane just before ovulation. Here we further develop the theoretical basis by applying laboratory observations of the various components of the fertilized egg in first-principle equations for egg specific gravity (ρ_egg_) followed by statistical validation. We selected Atlantic cod as a model animal due to the affluent amount of literature on this species, but also undertook additional dedicated experimental works. We found that specific gravity of yolk plus embryo is central in influencing ρ_egg_ and thereby the buoyancy. However, our established framework documents the effect on ρ_egg_ of the initial deposition of the heavy chorion material in the gonad prior to spawning. Thereafter, we describe the temporal changes in ρ_egg_ during incubation: Generally, the eggs showed a slight rise in ρ_egg_ from fertilization to mid-gastrulation followed by a gradual decrease until full development of main embryonic organs just before hatching. Ontogenetic changes in ρ_egg_ were significantly associated with volume and mass changes of yolk plus embryo. The initial ρ_egg_ at fertilization appeared significantly influenced by the chorion volume fraction which is determined by the combination of the final chorion volume of the oocyte and of the degree of swelling (hydrolyzation) prior to spawning. The outlined principles and algorithms are universal in nature and should therefore be applicable to fish eggs in general.

## Introduction

Buoyancy of fish eggs has been studied in a wealth of articles over the last decades [1], although the recent focus has shifted from actual measurements to understanding the molecular and physiological basis, in particular the active role of aquaporins during oocytic water uptake [2], [3]. In line with earlier studies [4], [5], [6], Govoni & Forward [1] presents in their review a formulation of Archimedes principles where egg buoyancy is set as a function of the density of ambient water and of the various egg components. Although this principle is well adopted in science, the terminology and thereby the construction of this commonly seen formula deviates from physics of today, e.g. ‘force’ is defined in grams. Modern principal equations were presented in Kjesbu *et al*. [7]. However, as only data on eggs in the gastrulation stage were presented, their study does not address explanatory factors behind the noticed change in egg buoyancy from fertilization to hatching, i.e. ‘incubation’ (see Jung *et al*. [8], [9] and references therein), the present topic of interest using Atlantic cod (*Gadus morhua*) as an object of study.

The introduction of the density gradient column by Coombs [10] for measuring specific gravity in fish eggs accelerated interest in buoyancy studies as it enabled recordings on live individual eggs at a precision and an accuracy far beyond any earlier methods. This high accuracy was a needed basis to model the relation between egg buoyancy and vertical distribution in the field [11], both for pelagic and mesopelagic fish eggs [12]. The first observations on specific gravity were focussing on differences across individual eggs (e.g. Solemdal & Sundby [13]) without taking into account possible changes in egg specific gravity throughout incubation. These individual differences were included in the model on vertical distribution of eggs by embedding a Gaussian distribution function on egg buoyancy balanced by the turbulent mixing [11]. Later it became evident that individual egg specific gravity also could vary systematically throughout incubation as exemplified on blue whiting eggs off the British Isles [14], Cape hake eggs off Namibia [15], and Atlantic mackerel and horse mackerel eggs off the British Isles [16] although these temporal changes in specific gravity was exceeded by the individual egg differences. The general feature of temporal change in the specific gravity throughout incubation, as demonstrated by Sundby *et al*. [15], was an initial increase after fertilization, and thereafter a decrease towards hatching. This development impacts the way the Cape hake eggs ascend from the spawning depth towards upper layers. In order to model with high accuracy how the vertical distribution of pelagic eggs is modified through incubation by the changes in the egg buoyancy in the presence of ambient turbulence, we need to both understand how the eggs are changing and how much. The present understanding of these changes throughout incubation, at least at the conceptual level [5], [7], is incomplete to explain systematic trends in buoyancy as noticed in the laboratory during the period of egg incubation [8], [17]–[19]. Craik & Harvey [4] showed that changes in water content of the egg influences the buoyancy, and such changes together with changes in composition of fat and proteins and changes through respiration could all act as factors to modify egg buoyancy onto the hatching (see Govoni & Forward [1] and references therein).

In the establishment of a true ρ_egg_ model, first-principle equations should be applied and these algorithms should reflect the fact that a fertilized fish egg typically consists of three parts: chorion, perivitelline space (PVS) and yolk plus embryo (Appendix: Eq. 1). Thus, the total egg mass is the sum of masses of these components, which can be expressed by their separate volumes (V’s) and specific gravities (ρ’s):
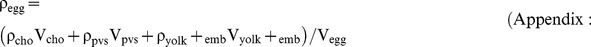
(Appendix: Eq.2)


Here the contribution of the chorion requires some special attention. This structure consists of proteins [20], which harden following egg fertilization [21]. While the chorion volume (V_cho_) may differ both among [22] and within individuals [7], the specific gravity of the wet chorionic material (ρ_cho_) is roughly constant, even between different species, but appears to be exceedingly high: e.g. 1.20 g cm^−3^ for Atlantic cod [7] and 1.18 g cm^−3^ for Atlantic halibut (*Hippoglossus hippoglossus*) [23]. In general, egg buoyancy (i.e. Δρ; Δρ = ρ_water_–ρ_egg_) in pelagic fish eggs is less than 0.003 g cm^−3^ [24], while ρ_water_ ranges from about 1.010 (brackish) to 1.028 g cm^−3^ (marine). As the chorion is permeable to the ambient seawater it is assumed that the specific gravity of the perivitelline space (ρ_pvs_) is identical to ρ_water_. Evidently, ρ_yolk+emb_ varies intraspecifically; the reported values are 1.017 g cm^−3^ and 1.008 g cm^−3^, respectively, for marine and brackish cod eggs [7]. In the latter case identical figures were found in another, independent study: 1.007–1.009 g cm^−3^ [25]. Thereby, the low ρ_yolk+emb_ counteracts the high ρ_cho_ to create the necessary hydrostatic lift of the egg. Both egg diameter and thereby egg volume (V_egg_) and chorion thickness and chorion volume (V_cho_) are considered constant throughout embryonic development, i.e., after the aforementioned initial short period of swelling and eggshell hardening that settles the volume of the egg. Therefore, also the sum of the volumes of the perivitelline space and the yolk plus embryo must be constant, i.e. V_pvs_+V_yolk+emb_ = constant, and hence changes in V_yolk+emb_ during development must result in compensating changes in V_pvs_ according to Eq. 3 (Appendix). Consequently, variability in ρ_egg_ during incubation, i.e. from fertilization to hatching, must be caused by changes in ρ_yolk+emb_. In regards to egg metabolism and physiological development, these changes in ρ_yolk+emb_ can be caused by two factors: (1) changes in small molecules including ions (free amino acids, NH_4_^+^, Cl^−^, K^+^ and Na^+^) and (2) changes in macromolecules (carbohydrates, proteins and lipids) [5].

During the work we encountered two hurdles: i) lack of sufficiently detailed algorithms of basic relationships and ii) missing or incomplete observations throughout incubation on key input variables for these equations. Hence we develop both the theoretical side (cf. Appendix) and a data collation programme undertaken under controlled conditions in the laboratory to provide values for the equations. The experimental study was performed to explore what *de facto* determines ρ_egg_, both at fertilization and during incubation, by including associated measurements of a series of relevant variables (cf. Appendix). These formulations show that V_yolk+emb_, ρ_yolk+emb_ and V_cho_ play important roles in defining ρ_egg_. We hypothesize that (1) initial variation in ρ_egg_ among individual newly spawned eggs from a single female, is due to differences in V_cho_, and that (2) trends in ρ_egg_ during embryonic development are caused by changes in ρ_yolk+emb_ and V_yolk+emb_. The value of ρ_yolk+emb_ (Appendix: Eq. 4) was achieved by consulting the new measurements. Hence, the present series of theoretical and practical approaches confirmed the role of ρ_yolk+emb_ in defining ρ_egg_.

## Materials and Methods

### Ethics statement

All experiments followed the ‘European Convention for the Protection of Vertebrate Animals used for Experimental and Other Scientific Purpose’ (ETS123: http://conventions.coe.int/treaty/en/treaties/html/123.htm). Key experimenters were properly certified in laboratory animal science (FELASA category C researchers) (http://www.felasa.eu/accreditation-boards/accreditation-board-for-education-and-training1/). Permissions for collecting eggs were granted by the commercial hatchery of the Marine Harvest Cod (MHC) and the governmental hatchery at Austevoll Research Station, the Institute of Marine Research, Norway. Both hatcheries were properly approved and registered by Norwegian authorities (The Norwegian Directorate of Fisheries; http://www.fiskeridir.no) and followed strict veterinary and husbandry guidelines. Our study was restricted to the use of fertilized eggs only. An initial document search clarified that such early life stages are not classified as ‘animals’ (ETS123: Part1-General Principles, Article 1,2a). Hence, in the present case no specific permission was needed from the Norwegian Animal Research Authority.

### Biological material

Experiments were performed on eggs provided by the commercial hatchery Marine Harvest Cod (MHC) for one winter (20^th^ Jan.–16^th^ Feb. 2009) and spring season (6^th^–31^st^ May 2009) and by the hatchery at the Austevoll Research Station, Institute of Marine Research (IMR), for one fall season (7^th^–31^st^ Oct. 2009). Female and male cod were stocked together in single spawning tanks (winter: 45 females and 50 males; spring: 9 females and 20 males; fall: 27 females and 15 males) and induced to spawn naturally by artificial day light rhythms. MHC broodstock cod were originally captured near the entrance of Sognefjord (50 km north-east from Bergen), and female cod age and body weight were on average 9 years and 8–15 kg for the winter experiment and 12–13 years and 15–25 kg for the spring experiment. The cod at IMR Austevoll were born and raised in captivity from wild specimens captured in Øygarden (20 km west from Bergen), and were 5 years in the present fall experiment. Data on the female body weight was not available. Sea water of spawning tanks in the two hatcheries was maintained at temperatures of 6–7°C and salinities of 34–35.

Eggs were collected three times with an interval of one week during one month each season (Batch1-Batch9; Table S1), and then transported from the hatcheries to the IMR laboratory in Bergen. Immediately after arrival the eggs were gently poured into small flow-through aquaria set up in a refrigerated room at 6°C and incubated until hatching. Sea water was pumped from the neighbouring fjord (∼100 m depth) and UV-filtered before entering the aquaria. Aquaria temperature varied between 7.6 and 8.3°C for winter, between 7.4 and 8.5°C for spring, and between 6.5 and 7.0°C for fall. Aquaria salinity was kept at 34–35 in the three seasons. The age of the egg was expressed in days post-fertilization (dpf) and specific description of egg developmental stages were according to Fridgeirsson [26]. Dead eggs sunken to the bottom were siphoned out every second day.

### Egg specific gravity

Egg specific gravity (ρ_egg_) was determined using a density-gradient column (Martin Instruments Co. Ltd, U.K.) set up in the refrigerated room (6°C). All preparation of density gradient, calibration, accuracy, stability and operation have in general been described by Coombs [10] and specifically for the present work by Jung *et al*. [8]. UV-filtered sea water, distilled water, and NaCl were used to make the low and high salinity stock solutions, which is needed to develop the density gradient in the column. Each of three columns of 80 cm height was filled by continuously graded sea water solutions at 7°C, and the completed density gradients ranged from 1.0125 g cm^−3^ at the surface of the column to 1.0325 g cm^−3^ on the bottom. At each experimental trial, five glass floats of known and calibrated densities (ρ_float_) were first introduced into the column from the top. The densities of the floats were originally reported at 23°C (or 18°C) with certified instruments in the Martin Instrument Co. (Three Crowns House, 160 Darlaston Road, Wednesbury WS10 7TA, UK). A density correction at 7°C was obtained according to the equation based on thermal expansion: ρ_float_ at 7°C = ρ_float_ at 23°C+(23–7)×ρ_float_ at 23°C×0.000028. After 1 h the glass floats had positioned at their levels of neutral buoyancy in the column and could be used as reference levels of specific gravity. Prepared eggs were gently inserted from the top of the column and located at their neutral buoyancies. The vertical centre position of the glass floats were read with a precision of 1 mm and formed the basis of linear regressions between column position and specific gravity (always r^2^>0.99). Due to the large number of eggs in the column and because the density gradient was set up to observe very small differences in egg specific gravity, we found it sufficiently accurate to count the eggs at every 10 mm vertical intervals. The eggs were first counted 30 min after the introduction into the column. The egg specific gravity, ρ_egg_, was derived using the regression equation (specific gravity precision between 10-mm intervals: ±2×10^−4 ^g cm^−3^; specific gravity resolution between 10-mm intervals: 2.5×10^−4 ^g cm^−3^; certified specific gravity precision of the calibrated floats: ±2×10^−4 ^g cm^−3^).

The measurements of ρ_egg_ were carried out in two different ways; one was ‘continuous measurements’ over time and the other was ‘time point measurements’ at a given stage of egg development: (1) The purpose of the former measurements was to track daily changes in specific gravity of the same eggs. About 50 eggs at early developmental stages of 2–3 dpf were submerged in a single column and continuously monitored for two weeks until hatching. ρ_egg_ was noted on a daily basis. Moribund and dead eggs in the column were counted every day for calculations of egg mortality rate and hatching rate from the initial number of eggs on the first day of the experiments. Moribund and dead eggs were apparent in that they were not transparent and with no sign of onward development from the previous day, or continuously sinking to the column bottom. Egg mortality rate was about 2–10% of the initial number of eggs. Hatching rate was about 2–8% at 12–13 dpf. (2) The latter measurements were aimed at studying co-variation of egg traits (see below) during development. About 100–300 eggs were introduced in another single column every second day. Sub-sets of these were recollected using a glass tube at assigned buoyancy layers (see below) for further scrutiny at different egg ages. Since the density gradient columns were newly made at each time point measurement occasion, the relatively large number of eggs did not affect the local oxygen conditions in the column. Due to the communal conditions of females and males in the same spawning tank, several egg batches were collected from different females on the same day. Generally, the individual differences in ρ_egg_ are considerably larger than the observed temporal changes of one single egg throughout incubation. Therefore, in measuring the temporal changes in ρ_egg_ we minimized the noise from the individual variations by selecting eggs at 2 dpf from a very narrow distribution (standard deviation: 0.0002 g cm^−3^) in the columns. Thus, egg specific gravity layers of different egg batches at 2–3 dpf were carefully selected based on the narrow distributions in the column (see standard deviations measured in each specific gravity layer, Table S1). During development one specific gravity layer was selected from the upper part and the other was from the lower part of the whole distribution.

### Egg wet weight, dry weight, and water content

Images of eggs sampled from defined buoyancy layers were taken by a digital camera (Olympus, SZX10) equipped on a stereomicroscope at 10×. For the measurements of egg wet weight, about 50 eggs were sub-sampled from the photographed eggs, rinsed carefully in distilled water for 60 sec to remove sea water ions from the perivitelline space [17], dried on a paper (15–20 sec), placed equally in five pre-weighed Nunc cryotubes (i.e. 10 eggs in each cryotube), and weighed on Cahn 25 Automatic Electrobalance (accuracy: ±1 µg). Immediately, the five groups of 50 eggs were frozen at −80°C for dry weight measurements. A few of the Batch5 and Batch6 samples were lost after being frozen. Egg dry weight was determined by a freeze drier (Christ Alpha 1–4 Loc-1m) operating at −50°C for 24 hrs. Egg wet weights and dry weights were calculated by dividing by the relevant number of eggs in each cryotube. Fraction of water content was determined as the difference between wet weight and dry weight divided by the wet weight. Eggs were separately preserved in 4% formaldehyde for the measurement of chorion thickness.

### Chorion thickness

Three eggs representing each specific gravity layer were treated individually for this procedure. First, the diameter of an egg was measured under a fluorescent microscope (Nikon, AZ-100) at 25×, and placed between two microscope slides on a soft cutting-off plate. The two glasses were placed side by side with a distance of an egg diameter, preventing the egg from moving during cutting process. The egg was sectioned into two halves under a stereomicroscope by a scalpel, and placed in a water-filled petri dish. Each half was observed under a fluorescent microscope at 400× during which the background light intensity was set at a grey level of 150–160. Chorion thickness (t) and egg diameter (2r) were measured in ImageJ and used for the calculation of chorion volume (V_cho_).

### Volume of yolk plus embryo and perivitelline space

With the captured egg images in ImageJ, individual yolk and embryo volume, V_yolk+emb_, was determined by an equation for an ellipsoid which is approximately closest to its actual form. After mid-gastrulation (∼4 dpf) the form of the embryo is starting to deviate from the ellipsoid shape. Hence, V_yolk+emb_ was not measured after ∼4 dpf. Perivitelline space volume (V_pvs_) was calculated as: V_pvs_ = V_egg_−V_cho_−V_yolk+emb_.

### Statistics

Statistical analyses were carried out using R version 2.10.0 and Statistica version 12 [27], [28]. The instrumental set-up of the density gradient column with a relatively high number of eggs within small vertical distances in the column did not allow for tracking and retrieving individual eggs. The measured egg characteristics were thus averaged across layers in the following statistical analysis. As stated above, specific gravity resolution between 10-mm intervals was 2.5×10^−4 ^g cm^−3^, while the certified specific gravity precisions of the calibrated floats are ±2×10^−4 ^g cm^−3^ implying that recording of vertical level of eggs within 10-mm intervals was near the possible specified precision of the method.

Analysis of covariance (ANCOVA) was used to compare slopes of regressions between continuous and time point measurements for trends in ρ_egg_ after the mid-gastrulation stage. Pearson correlation coefficient (*r*) was used to determine the strength of relationships between variables. In order to generate a predictive model of egg specific gravity we initially carried out a stepwise regression procedure with egg weight measures and different egg volume contributions (V_cho_, V_pvs_, and V_yolk+emb_) as independent variables to determine which of these variables significantly contributed to the determination of observed specific gravity. Only cases with no missing values were used. Subsequently a re-analysis was carried out with extra data left out at the initial analysis and a final multiple regression analysis was carried out with significant independent variables.

## Results

### Trends in egg specific gravity during incubation

**Figure 1:**
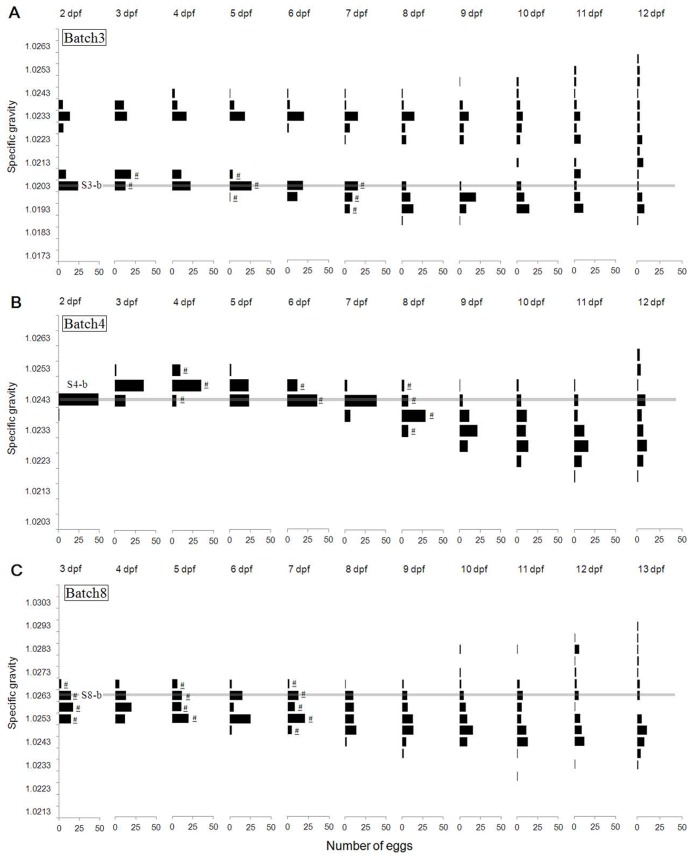
Ontogenetic changes of three seasons from 2–3 dpf until hatching (continuous egg specific gravity measurements). (A) Batch3, (B) Batch4, and (C) Batch8 represent winter, spring, and fall, respectively. Age of egg development is expressed in days post-fertilization (dpf). Horizontal lines refer to initial specific gravity of the selected layers (Batch3-b, Batch4-b and Batch8-b) at 2–3 dpf. Symbol of underlined # indicates the level of eggs used for slope tests in Table S2.

A similar pattern of temporal changes in egg specific gravity (ρ_egg_) was observed among the three seasons (Figure 1A (winter), Figure 1B (spring), Figure 1C (fall) and Figures S1–S3). In general terms, fertilized eggs showed a slight increase in ρ_egg_ until mid-gastrulation (3–4 dpf). Thereafter, the specific gravity gradually decreased until full development of main organs (9–10 dpf). Subsequently, ρ_egg_ suddenly increased just prior to hatching (11–13 dpf). The egg total incubation period was 12 days for winter and spring and 13 days for fall at 7°C. The egg distribution in a column was relatively narrow at early stages, but widened at later stages. Within the single egg batches the standard deviations were not significantly different from those of egg volume, chorion volume, and yolk volume measured from a single batch. Also such variations tended to be a bit higher at later stages of 10 and 12 dpf, but it does not hamper understanding of the general trend during development.

The decreasing trend in ρ_egg_ was similar between the two types of measurements (continuous *vs*. time point) for winter and spring, but different for fall (Table S2). Assuming that the environmental conditions of aquaria used for time point measurements were optimal for egg development, the lack of difference indicates that the eggs developed properly in the column in the winter and spring experiments.

### Initial changes in egg specific gravity as a function of perivitelline space and yolk volume dynamics

A slight increase in ρ_egg_ from 2 to 4 dpf was generally found in all samples. For examination of causes for this, specific gravity and volume changes in each component were tracked, including the chorion, by setting the mean ρ_cho_ to 1.20 g cm^−3^ and ρ_pvs_ identical to the ambient seawater (see Introduction). The specific gravity of yolk plus embryo (ρ_yolk+emb_) was estimated using Eq. 4 (Appendix) and appeared to be similar between 2 and 4 dpf. In terms of volume changes, V_pvs_ increased and V_yolk+emb_ decreased from 2 to 4 dpf while V_cho_ apparently was constant (Figure S4). Therefore, the noted increase in V_pvs_ and the comparable decrease in V_yolk+emb_ are presumed to cause the slight increase in the total ρ_egg_ from 2 to 4 dpf.

### Changes in egg characteristics during development

**Figure 2:**
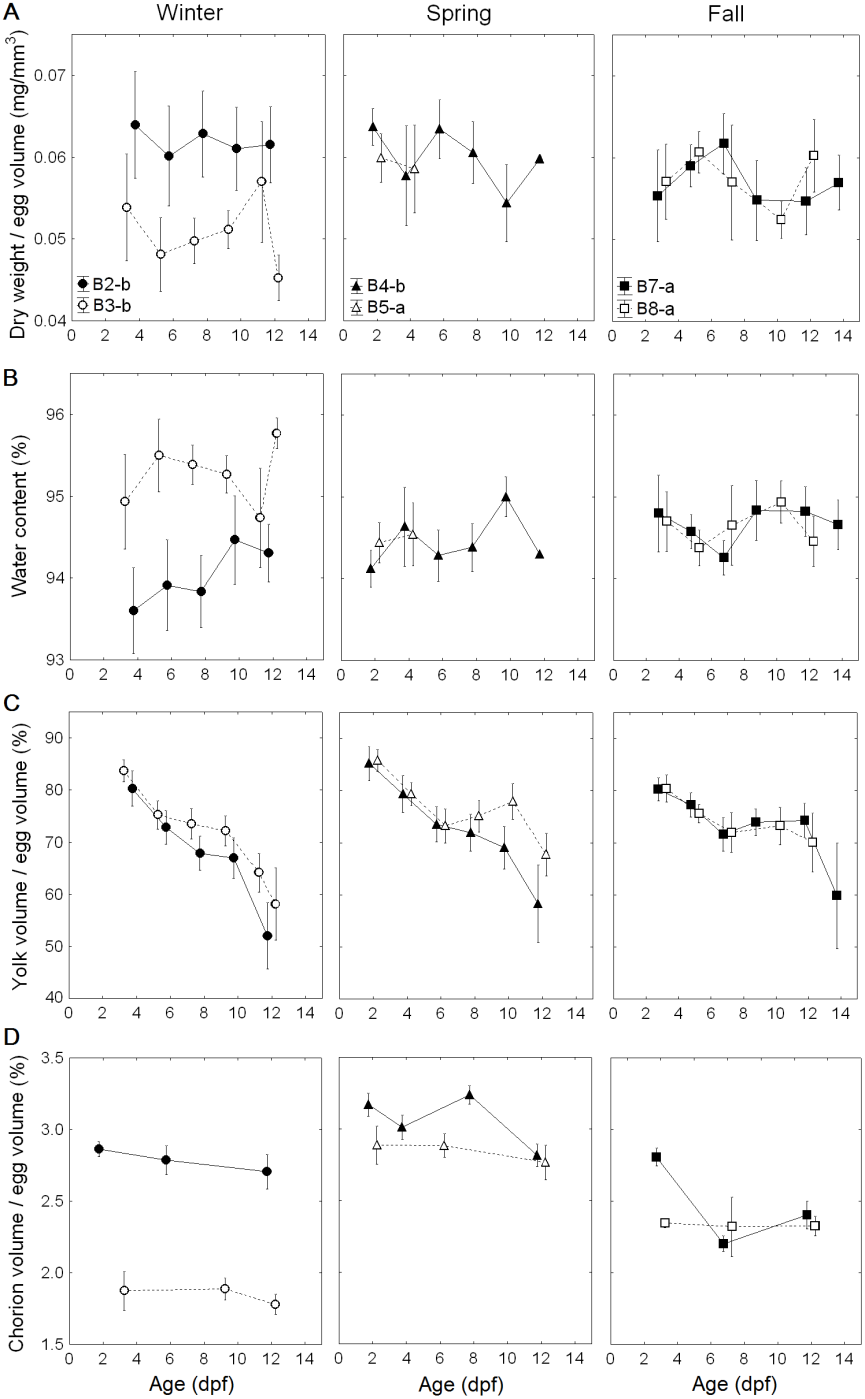
Ontogenetic changes in (A) egg dry weight/egg volume, (B) water content, (C) yolk volume/egg volume, and (D) chorion volume/egg volume. Selected buoyancy layers were Batch2-b and Batch3-b in winter, Batch4-b and Batch5-a in spring, and Batch7-a and Batch8-a in fall. As egg distribution overlapped with other layers of eggs, in particular after the latter half incubation, each egg component was divided by mean egg volume in order to avoid egg size effects except for the water content. Points refer to mean and one standard deviation.

Egg dry weight/egg volume did not show a systematic trend during development among the studied samples (Figure 2A). Only Batch4-b showed a significant negative correlation with age (*r* = −0.4, *p* = 0.043). Water content was positively correlated with age in two samples (Figure 2B), i.e. Batch2-b (*r* = 0.48, *p* = 0.015) and Batch4-b (*r* = 0.41, *p* = 0.040). Given the needed high precision of the measurements of dry weight and water content it is however, not surprising that trends are different for these measurements. V_yolk_/V_egg_ (where V_yolk_ in this figure is the yolk excluding the embryo volume) consistently showed a decreasing trend (all occasions, *p*<0.001) (Figure 2C). V_cho_/V_egg_ also revealed a negative correlation with age for Batch2-b (*r* = −0.55, *p* = 0.002), Batch4-b (*r* = −0.52, *p* = 0.001), Batch5-a (*r* = −0.40, *p* = 0.039) and Batch7-a (*r* = −0.61, *p* = 0.002) (Figure 2D). However, chorion itself became slightly thinner at 12 dpf compared to the initial value at 2 dpf for all samples (Batch2-b: 6.7 to 6.4 µm; Batch3-b: 4.7 to 4.3 µm; Batch4-b: 7.5 to 6.6 µm; Batch5-a: 7.2 to 6.6 µm; Batch7-a: 6.8 to 6.1 µm from 2 dpf to 12 dpf), except for Batch8-a where chorion thickness (t) was measured to become slightly thicker (5.5 to 5.8 µm). Since the data material in all was limited, no statistical test was done. If, however, the chorion really became thinner, a speculation of the cause could be associated with enzymatic dissolution of the chorion from the inside related to the hatching process.

### Statistical relationships between initial egg specific gravity and egg components

**Figure 3:**
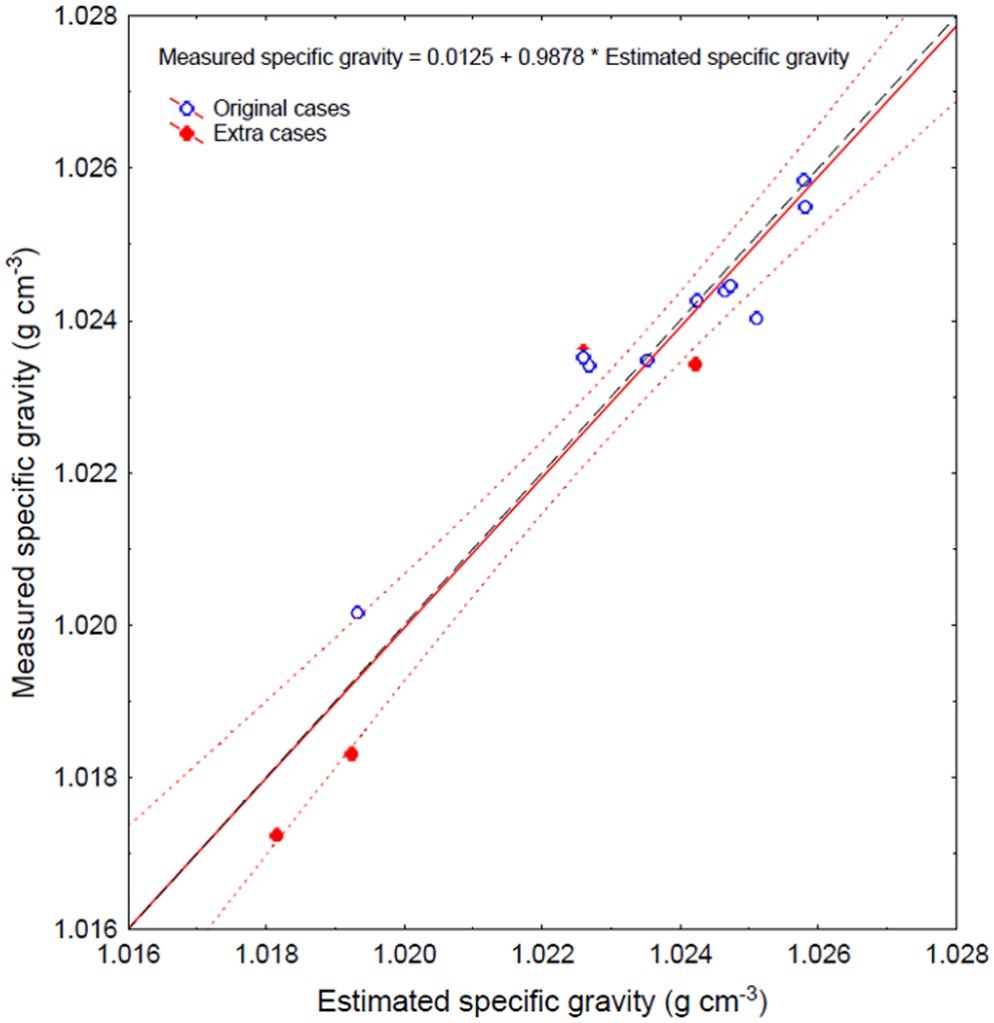
Estimated specific gravity (g cm^−3^) of cod eggs based on equation from multiple regression versus observed specific gravity from the same egg batches (solid line). Open symbols for original cases without missing variable values and filled symbols for extra data points included after the initial analysis. Dashed line represents 1∶1 line, and dotted lines represent 95% confidence regression bands.

Only the V_yolk+emb_ and V_cho_ contributed significantly to the specific gravity of the eggs (p<0.005, stepwise regression), while the V_pvs_ and wet- and dry weights did not. Based on the initial analysis, a re-analysis was carried out with cases that had been excluded due to missing values in non-significant independent variables. The residuals of the extra cases from the original relationship was not significantly different from 0 (t-test, p>0.05), and the re-analysis with all available data provided the following relationship based on the multiple regression: ρ_egg_ = 1.02697–0.01253×V_yolk+emb_+0.000278×V_cho_ (p<0.001, multiple regression, n = 14, r^2^ = 0.91) (Figure 3).

### Initial specific gravity of yolk plus embryo

**Figure 4:**
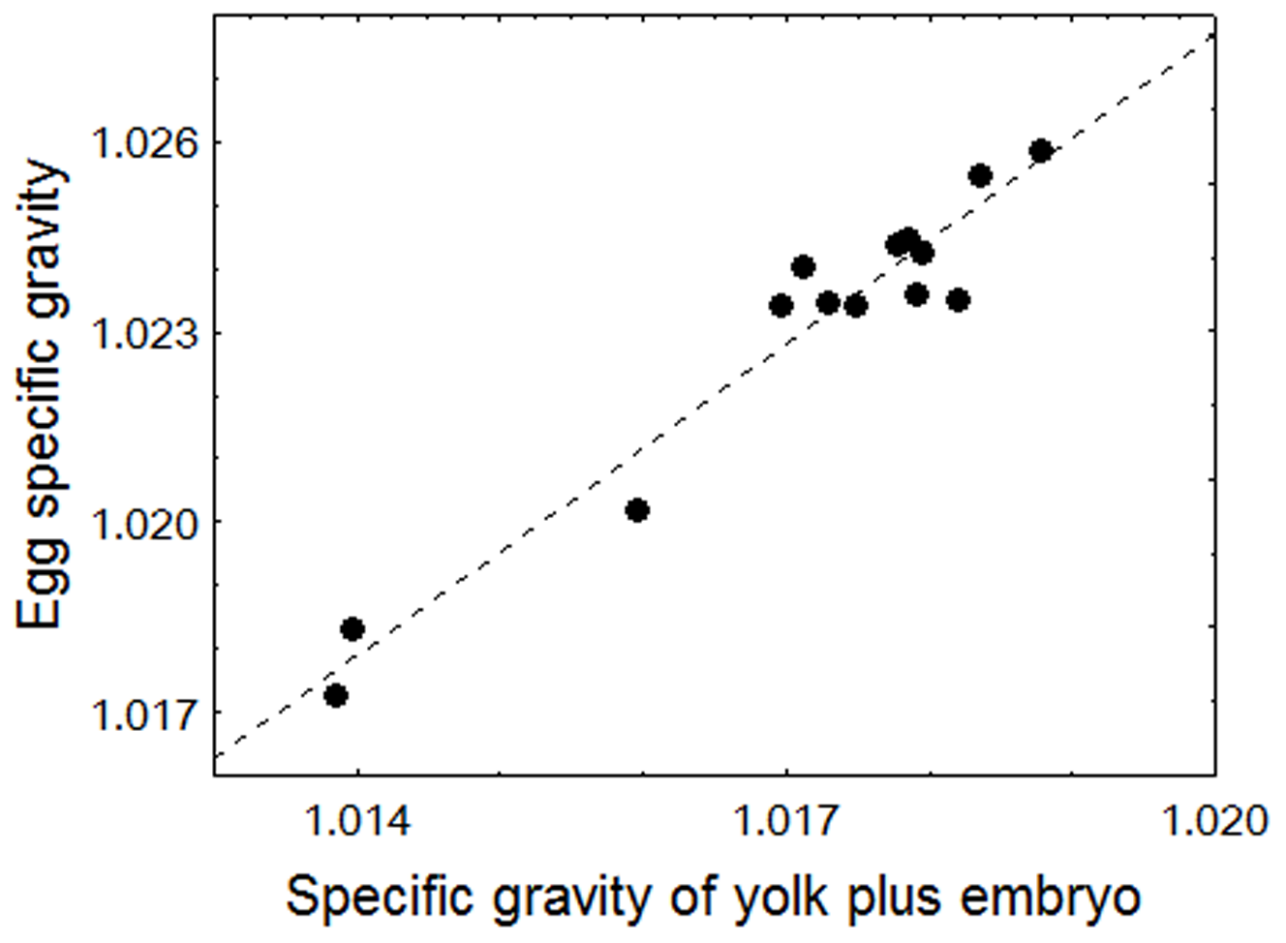
Relationship between egg specific gravity and specific gravity of yolk plus embryo at 2–3 dpf. The specific gravity of yolk plus embryo was estimated by Eq. 4 (Appendix).

By inserting our measured values of egg specific gravity (ρ_egg_), chorion thickness (t), egg diameter (2r), and yolk plus embryo volume (V_yolk+emb_) into Eq. 4, we calculated ρ_yolk+emb_ at 2–3 dpf. The relationship between ρ_egg_ and ρ_yolk+emb_ was highly significant. The linear regression was ρ_egg_ = 1.6326×ρ_yolk+emb_–0.6376 (r^2^ = 0.94) (Figure 4).

## Discussion

This study presents the first coherent explanation of the developmental and individual variability of egg specific gravity (ρ_egg_) for marine pelagic fish eggs based on first principles. It shows the relative contribution from the various components of the egg to the resulting egg specific gravity. Chronologically, the critical events in determining the changes in egg specific gravity are as follows: The first determinant starts in the ovary with the deposition of the heavy chorion material (M_cho_) associated with each oocyte [7]. The second determinant is associated with proteolytic cleavage and associated hydration of the yolk and the strong decrease in ρ_egg_ prior to spawning as addressed in the work by Craik & Harvey [4]. The third determinant is associated with the attained volume fraction of the neutrally buoyant perivitelline space (V_pvs_/V_egg_) as it manifests just after fertilization [4]. The fourth determinant is associated with the yolk plus embryo’s capability in osmoregulation that causes the egg initially to lose water and become heavier. Thereafter, with the increasing capability in osmoregulation, caused by the development of the embryo, the water content is restored and the egg becomes lighter again [29]. The fifth determinant is associated with the increasing specific gravity prior to hatching. The cause of this final egg specific gravity increase is not yet fully clear but a possible mechanism is considered at the end of the discussion. The sixth and final determinant is the hatching process that ends with the newly hatched larvae get rid of the heavy chorion and becomes considerably lighter.

As the early notable feature of ontogenetic changes, eggs increase their specific gravity up to 0.03% of the initial value from fertilization until mid-gastrulation (∼4 dpf). The slight increase in ρ_egg_ is attributable to the volume decrease in yolk plus embryo and the corresponding volume increase in PVS (Figure S4). The shrinkage of yolk plus embryo (V_yolk+emb_) without expansion of the chorion was also detected by Davenport *et al*. [30]. In addition, since egg osmoregulation is not effective until complete gastrulation [31], the passive water loss from the embryo to the hyper-osmotic ambient seawater is at maximum during the first four days [29], [32]. Thus, the decline in V_yolk+emb_, presumably resulting from osmotic water loss, may cause eggs to become slightly heavier at mid-gastrulation.

After 4 dpf, eggs generally show a gradual decline in ρ_egg_ until 10–11 dpf, but with a rapid increase at 12–13 dpf just before hatching. The rate of decrease in ρ_egg_ is 0.0002 g cm^−3^ day^−1^ (Table S2), implying that ρ_egg_ changes by 0.0014 g cm^−3^ from 4 to 11 dpf. During the same developmental stages, yolk volume fractions decrease with egg stages until hatching, while dry weight/egg volume, water content, and chorion volume fraction do not show consistent trends during development among the egg samples. Finn *et al*. [33] reported a similar trend of smoothly decreasing V_yolk_ in a single egg batch of Atlantic cod. They also found there were no significant changes in egg dry weight and water content throughout development. In a similar study for plaice (*Pleuronectes platessa*) eggs, wet weight, dry weight and water content were approximately constant until hatching [34]. Therefore, it seems that the changes in dry weight, water content, and chorion volume (V_cho_) do not properly explain temporal variability in ρ_egg_ during development.

Only changes in yolk plus embryo volume (V_yolk+emb_) and specific gravity (ρ_yolk+emb_) can cause the gradual decline in ρ_egg_, since egg diameter (2r), chorion mass (M_cho_), and chorion specific gravity (ρ_cho_) are approximately constant throughout development. Although a slight decrease in chorion thickness (t) prior to hatching was observed, resultant changes in M_cho_ are too small to significantly affect the ρ_egg_. The change in ρ_yolk+emb_ during development can, in turn, have two causes: (1) volume and mass changes in the yolk-sac linked to the water balance. Riis-Vestergaard [29] observed and calculated the change in yolk osmolarity in cod eggs raised in salinity of 34. He found a relatively rapid increase in yolk osmolarity from 340 at fertilization to around 410 at 3 dpf. Thereafter, the yolk osmolarity decreased to around 380 at 4 dpf and 320 at 10–12 dpf. The decrease in osmolarity from 380 to 320 corresponds to the decrease in ρ_egg_ of about 0.00142 g cm^−3^, i.e. very similar to our observation of 0.0014 g cm^−3^ calculated by the reduction rate. (2) The other is caused by volume and mass changes in embryo structural composition such as lipids, proteins, muscles and cartilage, as the embryo develops. Finn *et al*. [33] studied the respiration of cod eggs. The oxygen consumption increased by a factor of 10 from fertilisation to hatching and the ammonia excretion by a factor of 6. However, there was no clear trend in the development of the wet weight of the eggs although a reduction in the dry weight seemed to occur. Consumption of lipids will increase wet weight as water must replace this consumption, while consumption of proteins will decrease the total wet weight of the egg. In the subsequent calculation we, therefore, consider the loss of mass from the yolk plus embryo due to respiration and metabolism to be largely compensated by the water. It implies that ρ_yolk+emb_×V_yolk+emb_ = K, where K is constant. This means that a decrease in ρ_yolk+emb_ must be compensated by a corresponding increase in V_yolk+emb_. Under these assumptions, we can calculate the change in V_pvs_ (Appendix 5–8) derived from:
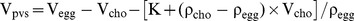
(8)


By inserting the observed reduction of ρ_egg_ (0.0014 g cm^−3^) throughout incubation into the equation, the calculated changes in V_pvs_ are only from 13.3 to 13.4% of the V_egg_ for an average egg (i.e. mean values of 2r, t, ρ_egg_, V_pvs_). This is in contrast to the much larger variation in V_pvs_ which varies from 17 to 22% of total egg volume during development [17]. Hence, we can conclude that the changes in ρ_egg_ and V_pvs_ throughout the first part of the incubation period onto 11 dpf are caused significantly by the changes in yolk osmolarity rather than the changes in embryo composition of proteins, bones, and muscles. Accordingly, the increase in osmolarity of yolk plus embryo after spawning results in loss of water and, consequently, reduced V_yolk+emb_, increased ρ_yolk+emb_, and subsequently increased V_pvs_ from fertilization to 4 dpf. Thereafter, the ρ_yolk+emb_ decreases since the decreasing yolk and the increasing embryo begin to actively take up water resulting in the increase in V_yolk+emb_ and the corresponding decrease in V_pvs_.

Our explanation for developmental variability in ρ_egg_ is consistent with egg osmoregulatory work [29]. Precursors of chloride cells are present at morula stage (∼2 dpf in the current study) and apparently functional after mid-gastrulation (∼4 dpf). Hence, the eggs regulate water loss and salt load by means of the chloride cells [31]. Restricted water loss has been found for cod [29], [32], and there was a positive relationship between buoyant eggs and sodium-potassium ATPase pump [9]. Mangor-Jensen [17] demonstrated that the mechanism for providing hydrostatic lift is the large volume of diluted tissue water located in the yolk and subdermal spaces. Accordingly, osmoregulatory ability of developing embryos is highly correlated with keeping proper egg buoyancy. Although the in-depth analyses of the changes in eggs throughout incubation are mainly demonstrated in cod, similar observations on changes in ρ_egg_ have been reported for other species where continuous measurements of egg specific gravity have been conducted. These include anchovy (*Engraulis encrasicolus*) [35], cape hake (*Merluccius capensis*) [15] and blue whiting (*Micromesistius poutassou*) [14].

Moreover, developmental changes in ρ_egg_ are also associated with catabolism of various energy substrates (carbohydrates, free amino acids (FAA), and lipids). For Atlantic cod eggs, FAA, which is markedly denser than ambient seawater, contributes 75% to aerobic catabolism [33]. The FAA is consumed for the purpose of respiration and protein synthesis, and 50% of the initial amount of FAA disappears before hatching [36]. Carbohydrates and lipids are present in low amounts during development as 0.3–0.7% and 10–13% of egg dry weight, respectively [33]. As a consequence, the substantial loss in FAA is likely to contribute to the decline in the yolk osmolarity throughout development.

Of egg structural materials, carbohydrates and proteins are denser than sea water and provide downwardly directed forces on the egg. In contrast, lipid and pure water are less dense and provide upward forces [4]. As such, egg specific gravity is initially determined by the balance of those materials in which proteins and water are regarded as major factors and carbohydrates and lipid as minor factors [4], [5], [6]. In the present study, chorion volume (V_cho_) at early egg stages consists from 2 (thickness: 3.8 µm) to 3.6% (thickness: 7.8 µm) of total egg volume (V_egg_) and significantly associates with ρ_egg_ ranging from 1.017 to 1.026 g cm^−3^. Similar positive relationship was observed for Baltic cod eggs in brackish water [37]. In their study, chorion thickness varied from 3 to 5 µm and positively correlated with ρ_egg_ ranging from 1.010 to 1.014 g cm^−3^. Kjesbu *et al.* [7] pointed out that two eggs with the same chorion volume (V_cho_) would have different egg specific gravity depending on egg diameter, because the ratio V_cho_/V_egg_ would change. Hence, a larger egg would have lower specific gravity than a smaller egg. Our results confirm the significant contribution of chorion volume to determining the initial egg specific gravity.

The V_pvs_ has been earlier reported to be about 20% of total egg volume [17], [30]. In the present study, the V_pvs_ largely varies from 9 to 18% at 2–3 dpf. Also the ρ_yolk+emb_ estimated in here ranges largely from 1.014 to 1.019 g cm^−3^, but mean ρ_yolk+emb_ is surprisingly similar to the previous measurement of Kjesbu *et al.* [7], 1.0170 *vs*. our estimate of 1.0171 g cm^−3^. Measurements on specific gravity on the hardened chorion material, ρ_cho_, from cod eggs Kjesbu *et al.* [7] show relatively constant values around 1.20 g cm^−3^. Also independent measurement on chorions from six Atlantic halibut eggs showed similar average value, i.e. 1.1842 g cm^−3^ [23]. Table 1 shows sensitivity of ρ_egg_ to the presently observed ranges of egg size, V_cho_, V_pvs_, and ρ_yolk+emb_. It appears from the table that the specific gravity of the cod eggs changes by 0.0063 g cm^−3^ across the range of egg size and V_cho_, i.e. the smallest eggs with thickest chorions are 0.0063 g cm^−3^ heavier than the extreme large eggs with thin chorions. In comparison, the extreme changes in ρ_yolk+emb_ cause ρ_egg_ to change by 0.0046 g cm^−3^, which is about 70% of the variation caused by the chorion volume fraction. It should be emphasized that total range of ρ_yolk+emb_ among individual eggs (i.e. 0.0046 g cm^−3^) is 3.3 times larger than the individual change in ρ_egg_ throughout incubation (i.e. 0.0014 g cm^−3^). We have not measured the initial degree of hydrolyzation of the proteins in the yolk of the present study. However, noting that the large difference in ρ_egg_ between Baltic cod and Norwegian Atlantic cod can partly be ascribed the variation in hydrolyzation of the yolk [17], [19] it is likely that the present observed (and smaller) differences in ρ_yolk+emb_ among individual eggs in Norwegian Atlantic cod could be explained by moderate individual differences in yolk hydrolyzation and, hence, the degree of egg swelling prior to spawning. While the chorion volume (V_cho_) is determined during the oocyte stage [38], the chorion volume fraction (V_cho_/V_egg_) is determined by subsequent hydrolyzation prior to spawning. The present findings on the variation range of the V_pvs_ are larger than earlier findings in the literature; 9–18% compared to 18–22% in Kjesbu *et al*. [7]. However, still with this larger range it has relatively small effects on the resulting changes in ρ_egg_: 0.0006 g cm^−3^, which is less than 10% of the variation in ρ_egg_ caused by the chorion volume fractions.

**Table 1:** Calculated influence of three egg components on egg specific gravity (ρ_egg_) for Atlantic cod based on observed ranges of the components.

	Differencein ρ_egg_ (g cm^−3^)	Range ofρ_egg_ (g cm^−3^)	D (mm)	t (µm)	V_cho_/V_egg_×100 (%)	V_pvs_/V_egg_×100 (%)	ρ_yolk+emb_ (g cm^−3^)
I	0.00626	1.02647	1.20	9.0	4.43	13.5	1.0171
		1.02021	1.50	3.7	1.47	13.5	1.0171
II	0.00063	1.02348	1.35	6.5	2.86	18.0	1.0171
		1.02285	1.35	6.5	2.86	9.0	1.0171
III	0.00464	1.02479	1.35	6.5	2.86	13.5	1.0188
		1.02015	1.35	6.5	2.86	13.5	1.0140

I: Influence of fraction of chorion volume in percent of total egg volume, (V_cho_/V_egg_)×100, by keeping the two other components constant and at average values. The observed upper value is based on smallest observed egg (D = 1.20 mm) [13] combined with the thickest observed chorion (t = 9.0 µm) [30] and the lower value is based on the largest observed egg (D = 1.50 mm) [13] and the thinnest observed chorion (t = 3.7 µm) (present study). II: Influence of the fraction of perivitelline space in percent of total egg volume, (V_pvs_/V_egg_)×100, by keeping the two other components at constant and at average values. III: Influence of specific gravity of yolk plus embryo, ρ_yolk+emb_, by keeping the two other components constant and at average values. The observed ranges of egg diameter, D, and chorion thickness, t, are based on the literature. All other ranges are based on the present data.

Some of the changes in ρ_egg_ during development may also be related to differences in the proximal composition of the yolk and embryo component, resulting in slight changes in ρ_yolk+emb_ from fertilization to hatching. For example, relatively higher free amino acid (FAA) content is expected in the yolk compared to the embryo [5], [36], with correspondingly higher relative protein content in the embryo as a result. Preliminary estimations of the ρ_yolk_ versus ρ_emb_ based on unfed newly hatched cod larvae, suggest that these differ by approx. 0.014 g cm^−3^ (own unpubl. results). During the initial few days after fertilization this difference is not expected to be of importance in the overall ρ_yolk+emb_ due to the relatively high mass of yolk relative to mass of embryo. However, as the volume of the embryo increases rapidly towards the end of incubation it is expected to contribute more to increasing the overall ρ_egg_.

In conclusion, based on our working hypotheses, the present study confirms that the initial ρ_egg_ is largely influenced by chorion volume fractions, and that the ontogenetic variability in ρ_egg_ during incubation is attributable to the volume and mass changes of yolk plus embryo. With the previous findings of water balance and metabolism during egg stages, we demonstrate that swelling of embryos by uptake of water and drop in yolk osmolarity are main causes of decreasing ρ_egg_ from mid-gastrulation to full development of main organs.

## Supporting Information

Figure S1 **Time point egg specific gravity measured in winter every second day during development.** Horizontal lines refer to initial specific gravity of the selected layers (Batch2-a and b, Batch3-a, b and c) at 2–3 dpf. Symbol of # indicates sampled levels for further analyses on egg composition shown in Figure 2 under the assumption that eggs initially having the same specific gravity as the Batch2-b and Batch3-b at 2–3 dpf would fall into the sampled levels with age. The underlined # in Batch3 indicates the level of eggs used for slope tests in Table S2.
(TIF)

Figure S2 **Time point egg specific gravity measured in spring every second day during development.** Horizontal lines refer to initial specific gravity of the selected layers (Batch4-a and b, Batch5-a and b) at 2 dpf. Symbol of # indicates pick-up levels for further analyses on egg composition shown in Figure 2 under the assumption that eggs initially having the same specific gravity as the Batch4-b and Batch5-a at 2 dpf would fall into the sampled levels with age. The underlined # in Batch4 indicates the level of eggs used for slope tests in Table S2.
(TIF)

Figure S3 **Time point egg specific gravity measured in fall every second day during development.** Horizontal lines refer to initial specific gravity of the selected layers (Batch7-a and b, Batch8-a and b) at 3 dpf. Symbol of # indicates pick-up levels for further analyses on egg composition shown in Figure 2 under the assumption that eggs initially having the same specific gravity as the Batch7-a and Batch8-a at 3 dpf would fall into the sampled levels with age. The underlined # in Batch8 indicates the level of eggs used for slope tests in Table S2.
(TIF)

Figure S4 **Initial changes in egg specific gravity caused by perivitelline space and yolk volume dynamics.** Left panel is changes in mean egg specific gravity and right panel is changes in mean volume fractions of yolk plus embryo, perivitelline space and chorion from 2 to 4 dpf, represented by spring time point measurements of (A) Batch4-b, (B) Batch5-a and (C) Batch6-a. Points refer to mean and one standard deviation.
(TIF)

Table S1 **Egg characteristics at 2–3 days post-fertilization (dpf) from time point measurements.**
(DOC)

Table S2 **Comparison of two types of measurements (continuous** ***vs*** **. time point).**
(DOC)

Appendix S1 **Theoretical equations for egg specific gravity containing (1) list of symbols in equations and (2) algorithms of specific gravity at fertilization and during development.**
(DOC)
