# Supplementary figures and images for: Experimental Parameterisation of Principal Physics in Buoyancy Variations of Marine Teleost Eggs
Source: PLoS One. 2014 Aug 14;9(8):e104089. doi: 10.1371/journal.pone.0104089 (PMC4133173; doi:10.1371/journal.pone.0104089)

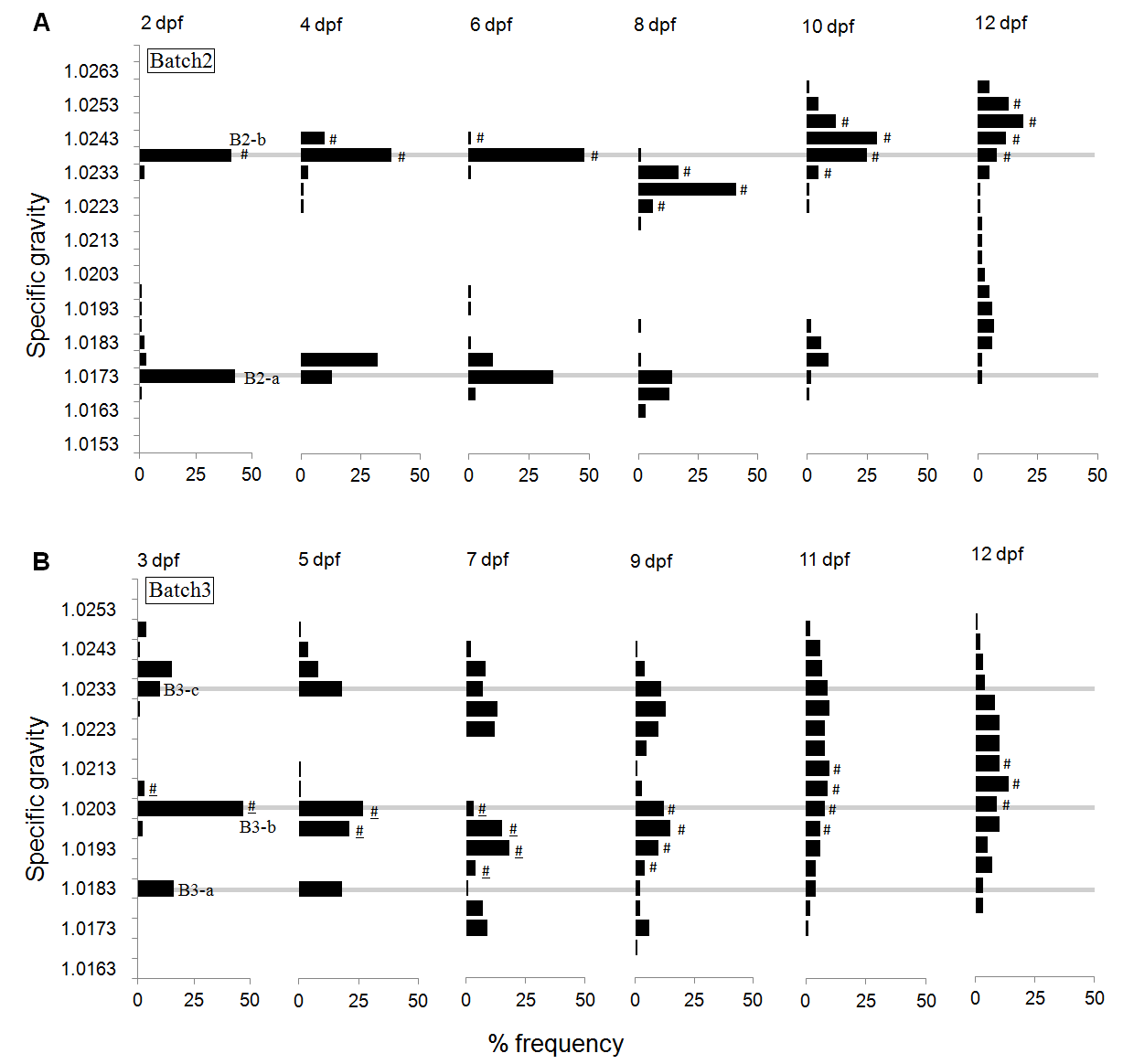

Supplement: Figure S1 — Time point egg specific gravity measured in winter every second day during development. Horizontal lines refer to initial specific gravity of the selected layers (Batch2-a and b, Batch3-a, b and c) at 2–3 dpf. Symbol of # indicates sampled levels for further analyses on egg composition shown in Figure 2 under the assumption that eggs initially having the same specific gravity as the Batch2-b and Batch3-b at 2–3 dpf would fall into the sampled levels with age. The underlined # in Batch3 indicates the level of eggs used for slope tests in Table S2. (TIF) [file pone.0104089.s001.tif]

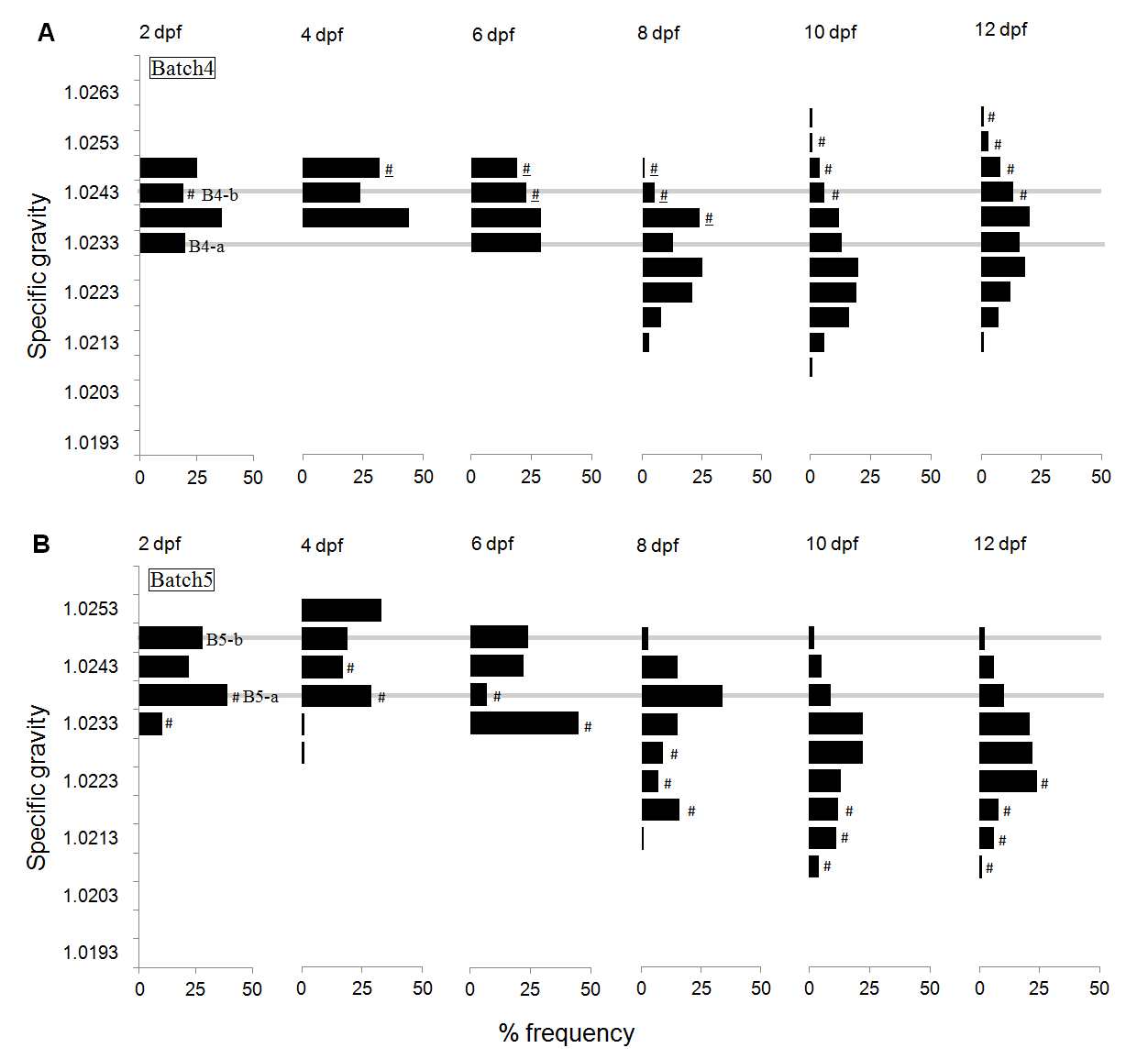

Supplement: Figure S2 — Time point egg specific gravity measured in spring every second day during development. Horizontal lines refer to initial specific gravity of the selected layers (Batch4-a and b, Batch5-a and b) at 2 dpf. Symbol of # indicates pick-up levels for further analyses on egg composition shown in Figure 2 under the assumption that eggs initially having the same specific gravity as the Batch4-b and Batch5-a at 2 dpf would fall into the sampled levels with age. The underlined # in Batch4 indicates the level of eggs used for slope tests in Table S2. (TIF) [file pone.0104089.s002.tif]

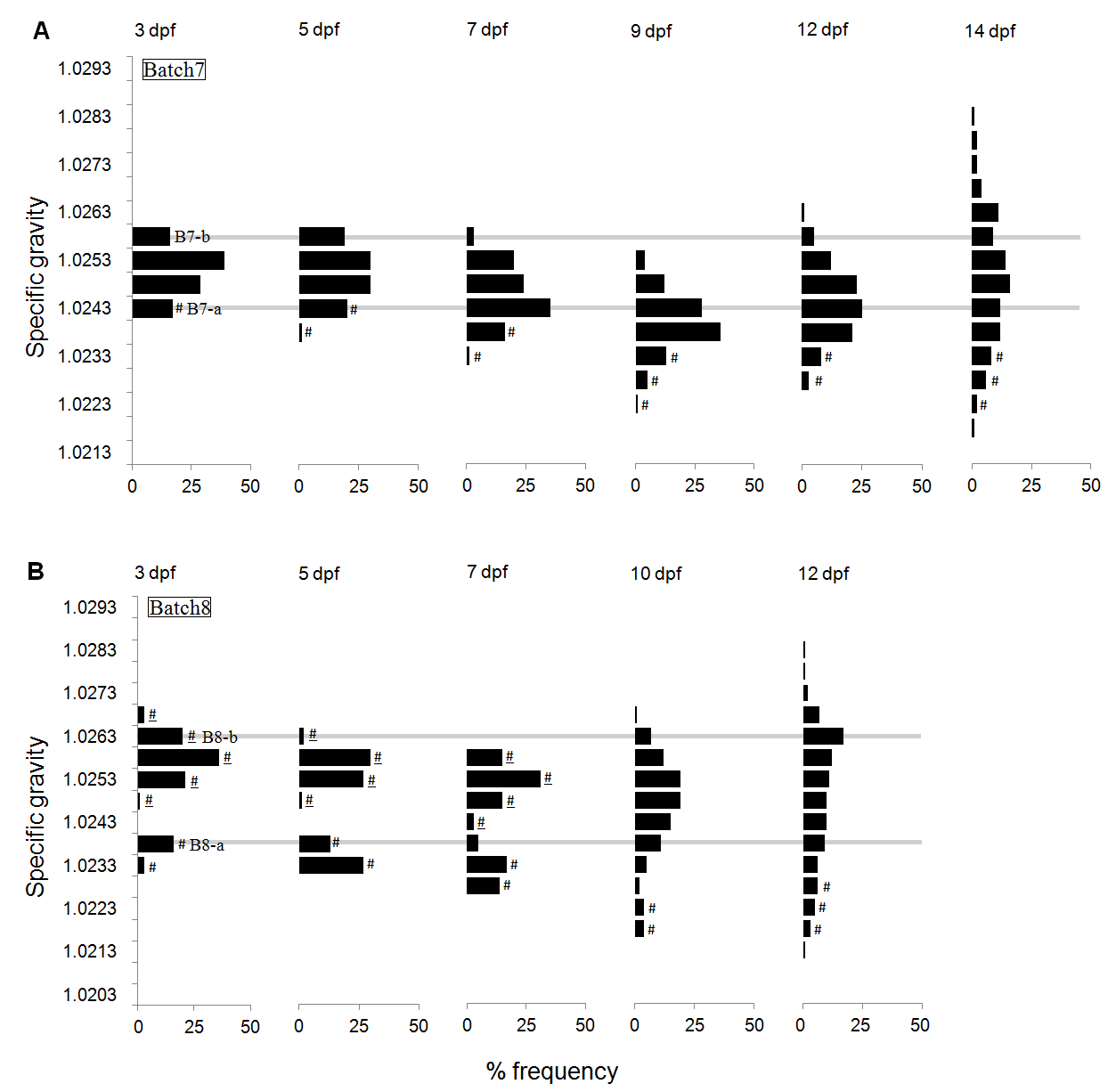

Supplement: Figure S3 — Time point egg specific gravity measured in fall every second day during development. Horizontal lines refer to initial specific gravity of the selected layers (Batch7-a and b, Batch8-a and b) at 3 dpf. Symbol of # indicates pick-up levels for further analyses on egg composition shown in Figure 2 under the assumption that eggs initially having the same specific gravity as the Batch7-a and Batch8-a at 3 dpf would fall into the sampled levels with age. The underlined # in Batch8 indicates the level of eggs used for slope tests in Table S2. (TIF) [file pone.0104089.s003.tif]

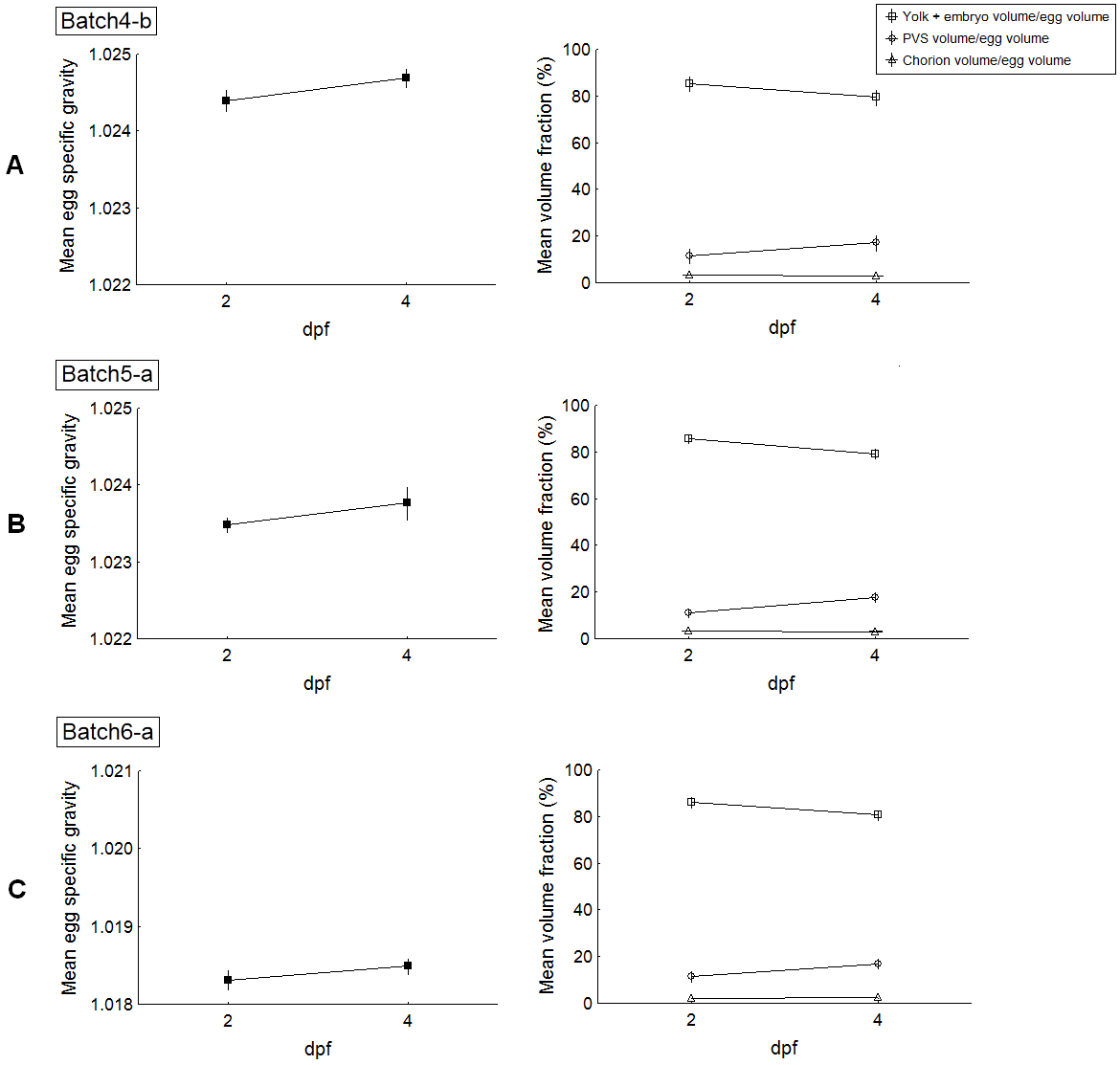

Supplement: Figure S4 — Initial changes in egg specific gravity caused by perivitelline space and yolk volume dynamics. Left panel is changes in mean egg specific gravity and right panel is changes in mean volume fractions of yolk plus embryo, perivitelline space and chorion from 2 to 4 dpf, represented by spring time point measurements of (A) Batch4-b, (B) Batch5-a and (C) Batch6-a. Points refer to mean and one standard deviation. (TIF) [file pone.0104089.s004.tif]
