# Supplementary material for: Experimental Parameterisation of Principal Physics in Buoyancy Variations of Marine Teleost Eggs
Source: PLoS One. 2014 Aug 14;9(8):e104089. doi: 10.1371/journal.pone.0104089 (PMC4133173; doi:10.1371/journal.pone.0104089)
Supplement: Table S1 — Egg characteristics at 2–3 days post-fertilization (dpf) from time point measurements. (DOC) [file pone.0104089.s005.doc]

**Table S1**. Egg characteristics at 2-3 days post-fertilization (dpf) from time point measurements.

|  |  |  |  |  |  |  |  |  |  |  |  |
| --- | --- | --- | --- | --- | --- | --- | --- | --- | --- | --- | --- |
| Hatchery | Season | Sample | Selected | dpf | Specific gravity | Salinity | Diameter | Dry weight | Water | Yolk volume | Chorion |
|  |  | no. | layer no. |  | n = > 50 | n = > 50 | n = 50 | n = 5 groups of 50 | n = 5 groups of 50 | n = 50 | n = 3 |
|  |  |  |  |  |  |  |  |  |  |  |  |
| MHC | Winter | Batch1 | Batch1-a | 3 | 1.0202 ± 0.0001 | 25.8 ± 0.1 | 1.43 ± 0.01 | (80) | (95.2) | 1.27 ± 0.03 | - |
|  |  |  | Batch1-b | 3 | 1.0234 ± 0.0003 | 29.9 ± 0.4 | 1.41 ± 0.02 | (130) | (91.8) | 1.21 ± 0.08 | 7.1 ± 0.2 |
|  |  | Batch2 | Batch2-a | 2 | 1.0173 ± 0.0001 | 22.1 ± 0.1 | 1.38 ± 0.01 | - | - | 1.20 ± 0.04 | 3.8 ± 0.2 |
|  |  |  | Batch2-b***** | 2 | 1.0236 ± 0.0001 | 30.2 ± 0.1 | 1.39 ± 0.01 | - | - | 1.25 ± 0.04 | 6.7 ± 0.2 |
|  |  | Batch3 | Batch3-a | 3 | 1.0183 ± 0.0001 | 23.4 ± 0.1 | 1.48 ± 0.02 | 82 ± 8.4 | 95.4 ± 0.4 | 1.46 ± 0.06 | - |
|  |  |  | Batch3-b***** | 3 | 1.0202 ± 0.0002 | 25.8 ± 0.2 | 1.43 ± 0.02 | 80 ± 10.0 | 94.9 ± 0.6 | 1.28 ± 0.05 | 4.7 ± 0.4 |
|  |  |  | Batch3-c | 3 | 1.0234 ± 0.0001 | 29.9 ± 0.2 | 1.47 ± 0.03 | 104 ± 5.5 | 94.3 ± 0.4 | 1.38 ± 0.09 | 6.9 ± 0.1 |
| MHC | Spring | Batch4 | Batch4-a | 2 | 1.0234 ± 0.0002 | 30.0 ± 0.2 | 1.41 ± 0.03 | 91 ± 5.7 | 94.4 ± 0.3 | 1.24 ± 0.06 | - |
|  |  |  | Batch4-b***** | 2 | 1.0244 ± 0.0001 | 31.2 ± 0.2 | 1.39 ± 0.03 | 89 ± 4.5 | 94.1 ± 0.4 | 1.19 ± 0.06 | 7.5 ± < 0.1 |
|  |  | Batch5 | Batch5-a***** | 2 | 1.0235 ± 0.0001 | 30.0 ± 0.1 | 1.42 ± 0.01 | 89 ± 4.4 | 94.4 ± 0.3 | 1.27 ± 0.04 | 7.2 ± 0.2 |
|  |  |  | Batch5-b | 2 | 1.0245 ± 0.0002 | 31.3 ± 0.2 | 1.36 ± 0.02 | 82 ± 5.6 | 94.2 ± 0.4 | 1.11 ± 0.04 | 7.3 ± 0.1 |
|  |  | Batch6 | Batch6-a | 2 | 1.0183 ± 0.0001 | 23.5 ± 0.2 | 1.43 ± 0.02 | - | - | 1.32 ± 0.05 | 4.9 ± 0.2 |
|  |  |  | Batch6-b | 2 | 1.0240 ± 0.0002 | 30.7 ± 0.2 | 1.42 ± 0.02 | (91) | (94.0) | 1.25 ± 0.05 | 7.8 ± 0.3 |
| IMR | Fall | Batch7 | Batch7-a***** | 3 | 1.0243 ± < 0.0001 | 31.1 ± 0.1 | 1.37 ± 0.02 | 75 ± 7.6 | 94.8 ± 0.5 | 1.10 ± 0.04 | 6.8 ± 0.2 |
|  |  |  | Batch7-b | 3 | 1.0255 ± < 0.0001 | 32.6 ± < 0.1 | 1.35 ± 0.02 | 76 ± 5.9 | 94.5 ± 0.4 | 1.06 ± 0.04 | 7.6 ± 0.3 |
|  |  | Batch8 | Batch8-a***** | 3 | 1.0235 ± 0.0001 | 30.1 ± 0.2 | 1.36 ± 0.03 | 75 ± 6.0 | 94.7 ± 0.4 | 1.06 ± 0.06 | 5.5 ± < 0.1 |
|  |  |  | Batch8-b | 3 | 1.0263 ± 0.0002 | 33.6 ± 0.2 | 1.29 ± 0.02 | 72 ± 2.6 | 94.1 ± 0.2 | 0.91 ± 0.06 | - |
|  |  | Batch9 | Batch9-a | 3 | 1.0241 ± 0.0001 | 30.9 ± 0.1 | 1.28 ± 0.02 | 66 ± 7.4 | 94.4 ± 0.6 | 0.89 ± 0.04 | - |
|  |  |  | Batch9-b | 3 | 1.0259 ± 0.0001 | 33.0 ± 0.2 | 1.33 ± 0.01 | 84 ± 6.2 | 93.6 ± 0.4 | 1.00 ± 0.04 | 7.4 ± 0.1 |

Selected layers showed narrow distributions distinguishable from other eggs in the column and specified in Figure 1 and Figures S1-S3. Egg specific gravity (g cm^-3^), egg diameter (mm), egg dry weight (µg), water content (%), yolk volume (µl), chorion thickness (µm) are shown as mean ± 1 standard deviation. N is the number of eggs used in each measurement. Numbers in parentheses are one value from one group of 10 eggs. Egg specific gravity was also expressed as neutral buoyancy in salinities at 7°C. Hyphens mean no data; ***** refers to selected layers for further analyses of ontogenetic changes.
