# Supplementary material for: Experimental Parameterisation of Principal Physics in Buoyancy Variations of Marine Teleost Eggs
Source: PLoS One. 2014 Aug 14;9(8):e104089. doi: 10.1371/journal.pone.0104089 (PMC4133173; doi:10.1371/journal.pone.0104089)
Supplement: Table S2 — Comparison of two types of measurements (continuous vs . time point). (DOC) [file pone.0104089.s006.doc]

**Table S2.** Comparison of two types of measurements (continuous *vs*. time point).

| **Season** | **dpf** | **Type (selected layer no.)** | **Regression equation** | **Slope test between continuous and time point measurements** | | |
| --- | --- | --- | --- | --- | --- | --- |
|  |  |  |  | **df** | ***F*-value** | ***p*-level** |
| Winter | 3, 5, 7 | Continuous (Batch3-b) | SG = -0.00016 × (dpf) + 1.0207 (r^2^ = 0.58) | 242 | 0.79 | 0.374 |
|  |  | Point (Batch3-b) | SG = -0.00018 × (dpf) + 1.0208 (r^2^ = 0.51) |  |  |  |
| Spring | 4, 6, 8 | Continuous (Batch4-b) | SG = -0.00020 × (dpf) + 1.0256 (r^2^ = 0.65) | 292 | 0.42 | 0.518 |
|  |  | Point (Batch4-b) | SG = -0.00021 × (dpf) + 1.0256 (r^2^ = 0.69) |  |  |  |
| Fall | 3, 5, 7 | Continuous (Batch8-b) | SG = -0.00005 × (dpf) + 1.0260 (r^2^ = 0.03) | 295 | 10.36 | **0.001** |
|  |  | Point (Batch8-b) | SG = -0.00015 × (dpf) + 1.0263 (r^2^ = 0.30) |  |  |  |

Trend of specific gravity (SG) decrease during incubation was estimated by linear regression model, and tested by slope differences between continuous and time point measurements. dpf refers to egg ages used for the regression model. The eggs used in the continuous measurements were the same eggs at the dpfs. The eggs used in the point measurements were the same egg batches but different eggs at each dpf.
