# Supplementary material for: Experimental Parameterisation of Principal Physics in Buoyancy Variations of Marine Teleost Eggs
Source: PLoS One. 2014 Aug 14;9(8):e104089. doi: 10.1371/journal.pone.0104089 (PMC4133173; doi:10.1371/journal.pone.0104089)
Supplement: Appendix S1 — Theoretical equations for egg specific gravity containing (1) list of symbols in equations and (2) algorithms of specific gravity at fertilization and during development. (DOC) [file pone.0104089.s007.doc]

**Appendix**

List of symbols in equations

M_egg_: mass of total egg

M_cho_: mass of chorion (eggshell)

M_pvs_: mass of perivitielline space

M_yolk+emb_: mass of yolk plus embryo

V_egg_: volume of total egg

V_cho_: volume of chorion

V_pvs_: volume of perivitelline space

V_yolk+emb_: volume of yolk plus embryo

ρ_egg_: specific gravity of total egg

ρ_cho_: specific gravity of chorion

ρ_pvs_: specific gravity of perivitelline space

ρ_yolk+emb_: specific gravity of yolk plus embryo

r: egg radius

t: chorion thickness

n: volume fraction of the yolk and embryo compared to the volume of the egg inside the chorion

Algorithms

Fish egg at fertilization consists of three separate components with different structures and specific gravities: (1) chorion (eggshell), which provides the protecting coat of the embryo, (2) perivitelline space, which functions as the medium for transfer of oxygen between the ovoplasm and the surrounding sea water, and (3) yolk plus blastodisc (a thin region of yolk-free cytoplasm), which is named as yolk plus embryo from fertilization until hatching in our study. Thus, total mass of the egg (M_egg_) is the sum of masses of the three components. That is:

M_egg_ = M_cho_+M_pvs_+M_yolk+emb_  (1)

Each of these components has their separate structures and specific gravities. Hence, specific gravity of the egg (ρ_egg_) can be expressed as:

(2)

Expressed in terms of egg radius, r, and chorion thickness, t, and volume fraction of yolk plus embryo, n, (inside the chorion) the relation can be written:

(3)

The specific gravity of the perivitelline space, ρ_pvs_, is equal to the egg specific gravity measured in the density gradient column, ρ_egg_. From Eq. 2 the specific gravity of yolk plus embryo is then:

(4)

During embryonic development, egg volume is the sum of volumes of chorion, perivitelline space, and yolk plus embryo as follows:

V_egg_ = V_cho_+V_pvs_+V_yolk+emb_  (5)

Conservation of mass is:

ρ_egg_V_egg_ = ρ_cho_V_cho_+ρ_egg_V_pvs_+ρ_yolk+emb_V_yolk+emb_ (6)

Assuming that mass of yolk plus embryo, M_yolk+emb_, is constant throughout incubation:

M_yolk+emb_ = ρ_yolk+emb_V_yolk+emb_ = constant = K (7)

By the deduction from equations (5) – (7), the volume of perivitelline space is:

(8)
